# Strand-specific RNA-Seq reveals widespread and developmentally regulated transcription of natural antisense transcripts in *Plasmodium falciparum*

**DOI:** 10.1186/1471-2164-15-150

**Published:** 2014-02-22

**Authors:** T Nicolai Siegel, Chung-Chau Hon, Qinfeng Zhang, Jose-Juan Lopez-Rubio, Christine Scheidig-Benatar, Rafael M Martins, Odile Sismeiro, Jean-Yves Coppée, Artur Scherf

**Affiliations:** Biology of Host-Parasite Interactions Unit, Institut Pasteur, Paris, France; CNRS URA2581, Paris, France; Cell Biology of Parasitism Unit, Institut Pasteur, Paris, France; INSERM U786, Paris, France; Plate-Forme Transcriptome et Epigénome, Département Génomes et Génétique, Institut Pasteur, Paris, France; Research Center for Infectious Diseases, University Wuerzburg, Josef Schneider-Str. 2/Bau D15, 97080 Wuerzburg, Germany; Institute of Infectious Diseases and Vaccine Development, Tongji University School of Medicine, 1239 Siping Road, Shanghai, 200092 China

**Keywords:** Directional RNA-Seq, *Plasmodium falciparum*, ncRNA, Antisense RNA, Natural antisense transcripts, Genes, 3′ UTR, Polyadenylation sites

## Abstract

**Background:**

Advances in high-throughput sequencing have led to the discovery of widespread transcription of natural antisense transcripts (NATs) in a large number of organisms, where these transcripts have been shown to play important roles in the regulation of gene expression. Likewise, the existence of NATs has been observed in *Plasmodium* but our understanding towards their genome-wide distribution remains incomplete due to the limited depth and uncertainties in the level of strand specificity of previous datasets.

**Results:**

To gain insights into the genome-wide distribution of NATs in *P. falciparum*, we performed RNA-ligation based strand-specific RNA sequencing at unprecedented depth. Our data indicate that 78.3% of the genome is transcribed during blood-stage development. Moreover, our analysis reveals significant levels of antisense transcription from at least 24% of protein-coding genes and that while expression levels of NATs change during the intraerythrocytic developmental cycle (IDC), they do not correlate with the corresponding mRNA levels. Interestingly, antisense transcription is not evenly distributed across coding regions (CDSs) but strongly clustered towards the 3′-end of CDSs. Furthermore, for a significant subset of NATs, transcript levels correlate with mRNA levels of neighboring genes.

Finally, we were able to identify the polyadenylation sites (PASs) for a subset of NATs, demonstrating that at least some NATs are polyadenylated. We also mapped the PASs of 3443 coding genes, yielding an average 3′ untranslated region length of 523 bp.

**Conclusions:**

Our strand-specific analysis of the *P. falciparum* transcriptome expands and strengthens the existing body of evidence that antisense transcription is a substantial phenomenon in *P. falciparum*. For a subset of neighboring genes we find that sense and antisense transcript levels are intricately linked while other NATs appear to be regulated independently of mRNA transcription. Our deep strand-specific dataset will provide a valuable resource for the precise determination of expression levels as it separates sense from antisense transcript levels, which we find to often significantly differ. In addition, the extensive novel data on 3′ UTR length will allow others to perform searches for regulatory motifs in the UTRs and help understand post-translational regulation in *P. falciparum*.

**Electronic supplementary material:**

The online version of this article (doi:10.1186/1471-2164-15-150) contains supplementary material, which is available to authorized users.

## Background

The protozoan parasite *Plasmodium falciparum* is responsible for the most lethal form of human malaria, leading to one million deaths annually. The clinical symptoms of malaria are caused by the intraerythrocytic stages of the parasite, which multiply inside the host’s red blood cells (RBCs). During the past decades much research has focused on understanding how gene regulation is achieved in *Plasmodium*. The publication of the *P. falciparum* genome sequence in 2002 [1] was followed by transcriptome analyses using microarrays [2, 3] and, more recently, high-throughput sequencing of cDNA (RNA-Seq) [4–7]. These analyses allowed determination of transcript levels for a large number of genes, helped to refine the original gene model and revealed a tight regulation of gene expression throughout the intraerythrocytic developmental cycle (IDC) of *P. falciparum*. Nevertheless, information regarding 5′ and 3′ untranslated regions (UTRs) and the degree of antisense transcription is still missing for most genes.

Transcription of non-coding RNA (ncRNA) is common in eukaryotes, for example more than 90% of the human genome is transcribed [8] whereas only ~1.5% of it encodes proteins. Based on their size and origin of transcription, ncRNAs are generally referred to as small (<200 nt) or long ncRNAs (lncRNAs, >200 nt) and intronic, genic or intergenic ncRNAs (transcribed from regions between CDSs). In addition, RNAs that are complementary to other endogenous RNAs, are referred to as natural antisense transcripts (NATs). Some NATs result from incomplete transcription termination of adjacent genes and appear to represent transcriptional noise. Other NATs are highly conserved and expressed in a developmentally regulated manner, with tissue-specific expression patterns [9], providing strong arguments for their regulatory roles in biological processes such as gene expression. Work in several organisms has established the regulatory role of NATs via the RNA-interference (RNAi) pathway, in which a specific group of NATs, called microRNA promote post-transcriptional gene silencing. This pathway has been described in a large number of organisms but it is notably absent from *Saccharomyces cerevisiae* and *P. falciparum*[10, 11]. However, even in organisms lacking a functional RNAi-machinery, NATs have been shown to play important roles in regulating gene expression [12] and many different mechanisms have been described by which NATs can regulate gene expression in an RNAi-independent manner. At the transcriptional level, NATs can cause transcriptional interference via physical collisions between transcribing polymerases [13]. Regulation at the post-transcriptional level can occur via the formation of sense-antisense duplex RNA. Nuclear retention of NATs is commonly observed and duplex formation may thus regulate gene expression by retaining mRNA in the nucleus. In the cytoplasm sense-antisense duplex formation has been shown to affect mRNA stability and translation efficiency (reviewed in [14]).

While most of the published transcriptome analyses in *P. falciparum* do not permit differentiation between sense and antisense transcripts, some studies provided a strand-specific analysis and revealed antisense transcription from multiple sites across the genome [7, 15, 16]. However, no comprehensive analyses of potential correlations between sense and antisense RNA transcript levels have been performed and a complete strand-specific transcriptome profile throughout the IDC of the parasite, covering even the highly AT-rich intergenic regions, is still lacking.

Recent advances in RNA-Seq technology, in particular the ability to perform strand-specific analyses (reviewed in [17]), the identification of a polymerase able to amplify even extremely AT-rich regions [18, 19] and an increase in sequence read length, have motivated us to combine these improvements into one RNA-Seq protocol and determine the genome-wide, strand-specific transcriptional profile for *P. falciparum.*

Using this protocol, we have generated strand-specific RNA-Seq libraries for parasites throughout the IDC as well as for separately purified nuclear and cytosolic RNA fractions resulting in the sequencing of close to 90 billion nucleotides. Our data demonstrate substantial antisense transcription for 24% of genes, developmental regulation of antisense transcripts, a strong bias of antisense transcription towards the 3′-end of genes and a complex picture of correlation between sense and antisense transcript levels. In addition, the coverage of highly AT-rich regions allowed us for the first time to map polyadenylation sites for 3443 asexual blood stage genes.

## Results

### Preparation of strand-specific RNA-Seq libraries

**Figure 1:**
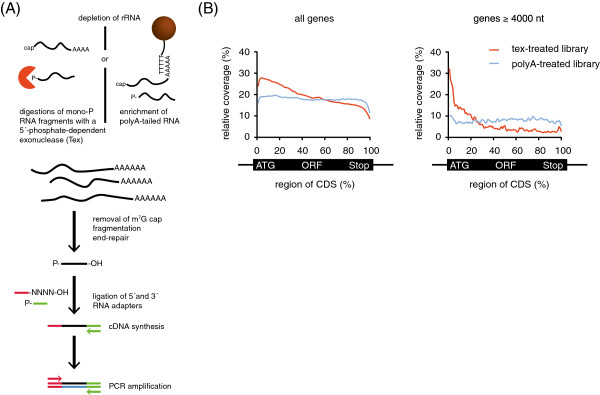
**Flowchart of strand-specific RNA-Seq library preparation. A)** Strand-specific sequencing libraries are prepared from total RNA depleted of rRNA by digestion of 5′-P-containing RNA fragments with a 5′-phosphate-dependent exonuclease (Tex) or by enrichment for polyadenylated mRNA. Subsequently, rRNA-depleted RNA is decapped, fragmented, dephosphorylated and re-phosphorylated to obtain RNA fragments with 5′-P and 3′-OH groups. Next, the RNA is ligated to 3′ and 5′-adapters, reverse-transcribed with a primer complementary to the 3′-adapter and the cDNA is PCR amplified for 12-16 cycles using KAPA HiFi polymerase. A recent analysis had compared amplification efficiencies of different polymerases for genomes ranging in GC-content from 67.7% (*Boretella pertussis*) to 19.3% (*P. falciparum*) and found KAPA HiFi (Kapabiosystems) to provide the most consistent results for AT-rich genomes [19]. **B)** Meta-gene coverage plots for libraries prepared from polyA-enriched and Tex-treated RNA. X-axis: scaled gene body, y-axis: scaled coverage.

To accurately determine RNA levels in the parasite, any biases that are inadvertently introduced during the preparation of cDNA libraries must be kept at a minimum while strand-specificity, coverage and sequencing depth should be maximized. Previously, a thorough comparison of different strand-specific RNA-Seq methods indicated that the least amount of ‘false’ antisense RNA was generated when libraries were prepared by using the ‘RNA-ligation method’ involving the sequential ligation of 3′-preadenylated and 5′-adapters to RNA followed by reverse transcription using a primer complementary to the 3′-adapter [17] (Figure 1A). Therefore, we adopted this method for use in *Plasmodium* although alternative strategies to generate strand-specific libraries may also be well suited [20].

**Figure 2:**
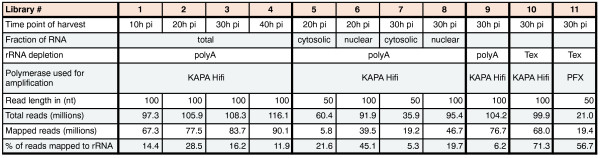
**RNA-Seq mapping statistics.** Libraries were prepared either from polyA-enriched (polyA) or Tex-treated RNA (Tex). DNA was amplified using the DNA polymerases KAPA Hifi (Kapabiosystems) or Platinum® Pfx (PFX, Invitrogen).

To increase the sequencing coverage of non-ribosomal RNA, we generated libraries from polyA-enriched RNA, unless indicated otherwise. We found polyA-enriched libraries to contain less rRNA than libraries prepared from RNA treated with the 5′-phosphate-dependent exonuclease (Tex) (Figure 2), an enzyme that specifically digests processed RNAs with a 5′-monophosphate end (e.g. rRNA). Furthermore, we noticed that the genome-wide coverage was substantially higher for libraries prepared from polyA-enriched libraries than for libraries prepared from Tex-treated RNA. This difference is probably a consequence of the lower percentage of rRNA found in libraries prepared from polyA-enriched RNA (Figure 2). Next we compared the distribution of sequence reads across CDSs for libraries prepared from polyA-enriched and Tex-treated RNA and noticed a strong bias towards the 5′-end in the Tex-treated library (Figure 1B). While this bias was not apparent for all genes, it was highly reproducible for individual genes and distinctly more pronounced in large genes. Therefore, to obtain more uniform sequencing coverage, we prepared all subsequent libraries from polyA-enriched RNA.

Using the RNA-ligation-based protocol, we prepared sequencing libraries from RNA extracted 10 h, 20 h, 30 h and 40 h post infection (p.i.) (n = 4), as well as from cytoplasmic and nucleic RNA of parasites harvested at 20 h and 30 h p.i. (n = 4). Combining the data from these libraries with the data from libraries prepared for protocol development (n = 3), our dataset consists of ~600 million mapped strand-specific reads derived from 11 libraries (Figure 2).

### Data coverage, level of strand-specificity and prevalence of NATs

Although the existence of NATs has been documented in *P. falciparum* based on SAGE, microarray and strand-specific high-throughput sequencing data [15, 21], the understanding of their genome-wide distribution is still incomplete due to the limited depth or uncertainties in the level of strand-specificity of the published datasets. Using strand-specific RNA-Seq data from ~30 million mapped reads, López-Barragán et al. observed NAT transcription in 312 coding genes [7]. To gain more insight into the genome-wide distribution of NATs, we took advantage of the unprecedented depth of our dataset (~600 million mapped reads, Figure 2) and combined the mapping results of 11 libraries to generate a coverage map. We also re-analyzed the López-Barragán et al. dataset (pooled from 4 strand-specific libraries in [7]) in parallel for comparison of coverage and level of strand-specificity.

Using the combined data from 11 libraries, we detected transcription of 78.3% of the *Plasmodium* genome (≥5-fold coverage, Additional file 1: Figure S1). Keeping all parameters constant, re-analysis of the López-Barragán et al. dataset indicated transcription of ~39.5% of the *Plasmodium* genome. Most of the transcribed genomic positions detected in the López-Barragán et al. dataset were also detected in this study (Additional file 2: Figure S2A), suggesting both datasets are consistent and that our dataset represents a substantially deeper coverage.

Next, we tried to determine the level of strand-specificity of our datasets compared to that generated by López-Barragán et al. Previously, a globally positive correlation between sense and antisense transcripts has been used as an indicator for potential presence of artifactual antisense transcripts [22, 23]. This approach is based on the fact that the observed antisense transcripts could be derived from the sense transcript during library preparation, e.g. incomplete second-strand cDNA digestion in dUTP method [7], spurious synthesis of second-strand cDNA [22], or other unidentified sources of artifacts. While the correlation in our dataset was close to 0 (Pearson’s correlation = 0.057, P < 0.01), we observed a strong positive correlation for the López-Barragán et al. dataset (Pearson’s correlation = 0.82, P < 0.01, Additional file 2: Figure S2B), suggesting a relatively higher level of strand-specificity of our dataset. Likewise, the ratios of sense to antisense reads differ significantly between our (1 antisense to 328 sense reads) and the López-Barragán et al. dataset (1 to 11.25). The relatively lower level of strand-specificity in López-Barragán et al. dataset maybe due to the imperfect second-strand cDNA digestion in dUTP method as the authors mentioned [7], comparing to the RNA ligation method used in this study. López-Barragán et al. thus avoided false positives in NAT identification by applying stringent cutoffs on both proportion of antisense read (>70%) and antisense read number (>150) [7], which may have underestimated the prevalence of NATs.

To estimate the prevalence of NATs in *Plasmodium*, we established two sets of thresholds with different stringency (see Methods for details). Our data indicate that between ~24% (n = 1247, stringent thresholds) and ~45% (n = 2389, relaxed thresholds) of all coding genes (n = 5284) are overlapping with NATs (Additional file 3: Table S1). In the following sections, unless mentioned otherwise, we restricted our analyses to the genes with NATs defined at stringent thresholds (n = 1247).

### Transcription of NATs is pervasive and developmentally regulated

Numerous mechanisms have been described by which antisense RNA can regulate gene expression even in the absence of a functional RNAi-machinery. Common to these mechanisms is the ability of the respective organism to regulate antisense transcription independently of the complementary sense transcription.

Previously, the distinct regulation of mRNA levels during the IDC has been described [2]. The IDC of *P. falciparum* takes 48 h to complete, thus, to evaluate developmental regulation of NATs across the IDC, we compared patterns of sense and antisense expression in parasites at evenly spaced intervals (10 h, 20 h, 30 h and 40 h p.i.).

To determine whether transcription of NATs is developmentally regulated, we searched for changes in NAT levels across the IDC. Using the software package EdgeR, we observed significant changes (FDR <0.05) in antisense transcript levels for 357 of the 1247 genes with NATs in at least 1 of the 6 non-redundant time point pair comparisons. For 125 genes with NATs we observed significant changes in antisense transcript levels in at least 2 of the 6 non-redundant time point pair comparisons (see Methods for details). These findings suggest that at least ~10% of NATs are regulated during the IDC (Additional file 3: Table S1 and Additional file 4: Tables S2).

**Figure 3:**
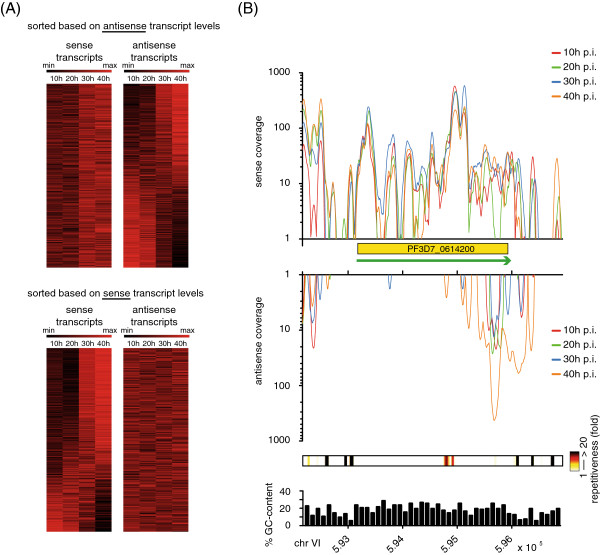
**Antisense transcription is abundant and developmentally regulated. A)** Heat map of genes containing sense and antisense transcripts throughout the IDC (n = 2389, NATs defined with relaxed threshold) sorted based on antisense RNA trends (top panel) and sense RNA trends (bottom panel). **B)** Coverage plot for a gene containing developmentally regulated NATs. Yellow box represents CDS and green arrow indicates direction of transcription.

Subsequently, we investigated whether the changes in sense and antisense transcript levels between times points are correlated and observed no global correlation (Additional file 5: Figure S3). Thus, our data suggest that transcription of NATs does not exert a globally positive or negative effect on the transcription of its sense counterpart. To further investigate this observation visually, we sorted the genes based on patterns of antisense expression levels throughout the IDC and plotted the antisense expression heat map (n = 2389, relaxed threshold), and observed a distinct life cycle-specific expression pattern of antisense transcripts. Then, we re-plotted the heat map using sense expression levels while keeping the gene order sorted using antisense expression levels. In agreement with the above observation, no apparent pattern was observed (Figure 3A, top panel). It should be noted that sorting the genes based on sense transcript levels confirmed the previously observed regulation of sense transcript levels [2] (Figure 3A, bottom panel). Thus, both sense and antisense transcript levels are regulated during the IDC but regulation is not synchronous. An example of developmentally regulated antisense transcript levels is shown in Figure 3B.

### Transcription of NATs is biased towards the 3′-end of genes

**Figure 4:**
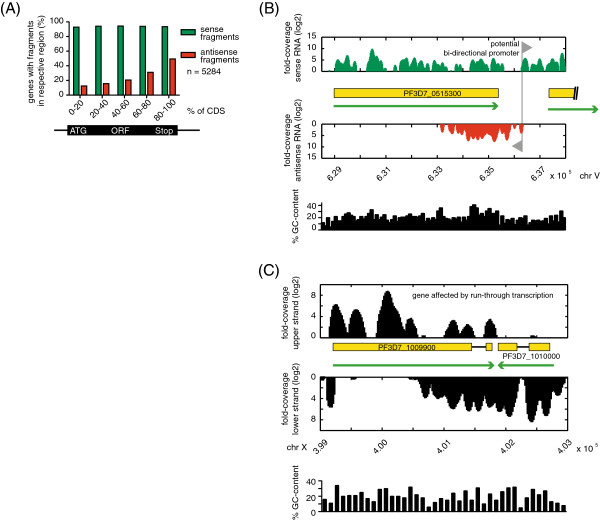
**Antisense transcription is strongly enriched at the 3′-end of CDSs. A)** Averaged coverage of antisense and sense RNA levels for all annotated genes. **B)** Sense (green) and antisense (red) coverage for a representative gene (PF3D7_0515300). Green arrows indicate direction of transcription and yellow boxes represent CDSs. The grey triangles mark the site of a putative bi-directional promoter. **C)** Coverage plot for a representative gene (PF3D7_1009900) likely to be affected by run-through transcription from a neighboring gene (PF3D7_101000). Green arrows indicate direction of transcription and yellow boxes represent CDSs.

**Figure 5:**
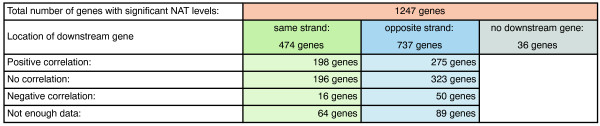
**Correlation of NATs with orientation of downstream genes.** Figure summarizing the correlation of NAT levels of a reference gene (stringent) with the mRNA levels of the gene located downstream.

**Figure 6:**
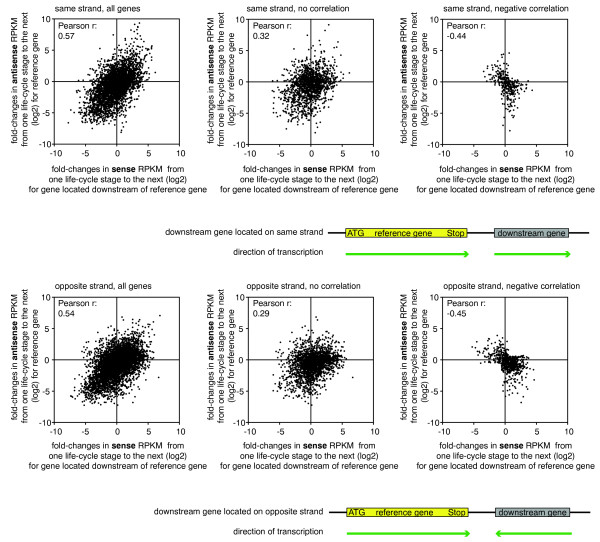
**NAT levels often correlate with expression of downstream genes.** Scatter plots showing fold-changes of anti-sense RPKM of a reference gene versus fold-changes of sense RPKM for the gene located downstream of the reference gene located on the same strand (top panel) or opposite strand (bottom panel). Time-point comparisons are plotted if the reference gene and downstream gene had significant anti-sense RPKMs and sense RPKMs for at least two time points. Up to six non-redundant time-point comparisons are plotted per gene.

To further characterize the newly identified NATs, we mapped antisense transcript levels across the CDSs of all genes and observed a striking enrichment of antisense transcripts at the 3′-ends of genes (Figure 4A). It has been suggested that NATs could be a consequence of run-through transcription or transcription initiation of bi-directional promoters from adjacent genes (for example see Figures 4B-C) [24, 25]. To determine if *P. falciparum* contains NATs whose expression is regulated independently from that of adjacent genes, we investigated the correlation between the changes in mRNA levels of the downstream genes and the changes in antisense transcript levels of the corresponding upstream genes during the IDC. We reasoned that, should antisense transcription be a consequence of active transcription of the downstream gene, changes in antisense transcript level across the IDC should be positively correlated to those of the downstream mRNA. To this end, we performed 6 non-redundant time-point pair comparisons for each gene (see Methods and Additional file 6: Table S3) and observed a positive correlation between the change of antisense transcript levels of one gene and that of mRNA levels of the gene located downstream (Figure 5). The observed correlation was independent of the orientation of the downstream gene, suggesting that both run-through transcription and bi-directional promoter activity contribute to the observed antisense transcription in *Plasmodium*. However, while we observed a positive correlation for 473 of 1247 genes with overlapping NATs (37.9%), for 66 genes (5.3%) we observed a negative correlation and for 519 genes (41.6%) no clear correlation (Figure 6). For 189 (15.2%) genes we did not have enough data points or no gene was located downstream. Among the NATs whose transcription did not correlate with the transcription of adjacent genes (n = 519 of 1247), i.e. NATs that appear to be independently regulated, we found ~13% (n = 67 of 519) to be developmentally regulated (for details see Methods, Additional file 3: Table S1 and Additional file 6: Tables S3). Taken together, these findings indicate that while run-through transcription and bi-directional promoter activity are likely to contribute to the observed antisense transcription, a significant number of NATs seems to be regulated independently of mRNA transcription.

### NATs do not accumulate in the nucleus or correlate with mRNA levels

To understand the role of antisense transcription in *Plasmodium*, we sought to determine whether our data provide evidence to support the known mechanisms for antisense-mediated regulation of gene expression.

Collisions between RNA polymerases transcribing the sense and antisense strands have been described to interfere with the transcription of mRNAs as shown in *Escherichia coli*[26] and *S. cerevisiae*[27]. If antisense transcription has a negative impact on sense transcription in *Plasmodium*, an increase in antisense levels from one time point to the next should correlate with a decrease in sense levels and vice versa. However, as mentioned above, comparison of changes in sense and antisense levels during four time-points showed a correlation close to 0 (Pearson’s correlation = 0.07, Additional file 5: Figure S3).

Alternatively, duplex formation between sense and antisense RNA molecules can result in nuclear retention of antisense transcripts and modulate gene expression. If antisense transcripts played a role in retaining sense transcripts in the nucleus, an enrichment of antisense transcripts in the nucleus would be observed. Several studies have reported nuclear localization of ncRNA in *P. falciparum*[28–30]. Thus, even though polyadenylated NATs are not commonly enriched in the nucleus, and our sequencing libraries were enriched for polyadenylated RNA, we examined the possible nuclear enrichment of antisense reads in genes that showed significant levels of antisense transcription but are not likely to be affected by run-through transcription from neighboring genes (listed in Additional file 3: Table S1). We did not observe a significant enrichment of antisense reads in these genes (n = 198) in the nucleus against both the total and the cytosolic fractions at 20 h p.i., while ncRNA from telomere-associated repeated elements were enriched in the nuclear fraction (Additional file 7: Figure S4).

A final mechanism we considered was antisense-mediated translational inhibition in which case overexpression of antisense transcripts leads to a reduction of proteins but not to reduction of sense RNA levels [31]. To this end, we correlated our RNA-Seq data with previously published stage-specific proteomic datasets [32]. As observed by Le Roch et al., we saw a positive correlation between mRNA abundance (in RPKM (reads per kilobase per million)) and protein abundance (in peptide counts per kilobase), thus an increase in sense RPKM correlates with an increase in peptide counts per kilobase for a given gene (Pearson’s correlation = 0.59 to 0.66 in 4 time points; Additional file 8: Figure S5A). To evaluate the global effect of antisense transcripts on translation, we investigated the correlation between antisense transcript levels (in antisense RPKM) and translation efficiency (in peptide counts per sense RPKM). However, for all four time points the correlation was close to 0 (Pearson’s correlation = -0.02 to 0.16 in 4 time points; Additional file 8: Figure S5B), suggesting that the level of antisense transcription has no global effect on translation efficiency.

### Mapping of sense and antisense polyadenylation sites

The sequencing depth of our dataset combined with the use of a polymerase better suited for the amplification of extremely AT-rich regions than previously used enzymes (Additional file 9: Figure S6 and [19]), allowed us to map for the first time polyadenylation sites (PAS) for *P. falciparum*. Despite the importance of 3′-UTRs in the regulation of gene expression, the genome-wide characterization of 3′-UTRs in *Plasmodium* lags far behind that of coding sequences. To determine PASs and to address the question of whether the observed antisense transcripts are polyadenylated, we used the combined RNA-Seq data from all 11 libraries to map PASs on a genome-wide scale. Due to the high abundance of coding mRNAs compared to antisense RNA (Additional file 3: Table S1), we expected most of the identified PASs to correspond to coding mRNAs and only a small fraction to correspond to NATs.

**Figure 7:**
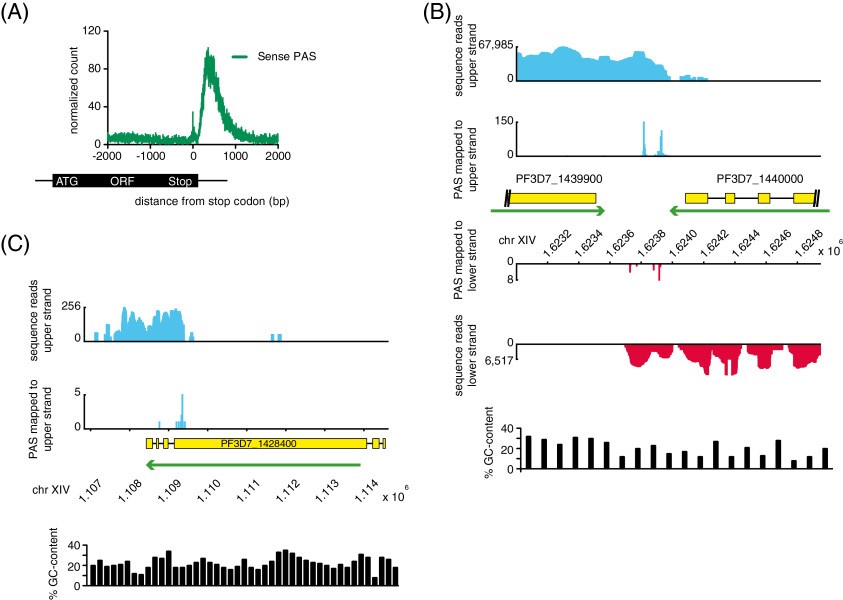
**Mapping of polyadenylation sites. A)** Distribution of mapped sense PASs (green), distance (bp) relative to the corresponding stop codon. **B)** Distribution of sequence reads and PASs mapping to the upper (blue) and lower (red) strand. Green arrows indicate direction of sense transcription for the corresponding CDS and yellow boxes mark CDSs. **C)** Distribution of antisense sequence reads and antisense PASs mapping to the upper strand (blue). The green arrow indicates the direction of sense transcription for the corresponding CDS and the yellow box marks the CDS.

To map genuine PASs and to avoid false positive assignments derived from internal polyA stretches or sequence reads of low quality, we followed a set of previously published criteria [33] (see Methods). To assess whether the identified PASs could represent genuine PASs, we plotted the occurrence of PASs around stop codons of all genes (Figure 7A). Most of the identified PASs fell within 1000 nt downstream of the stop codon of genes, corresponding to the expected sites of polyadenylation and therefore validating our approach. As microheterogeneity in PAS selection is well documented [34], we grouped individual PASs located in close proximity to each other into so-called ‘PAS-clusters’ with a maximum width of 20 nt. Setting the maximum 3′-UTR length to 2000 nt, we were able to assign 6678 PAS-clusters to 3443 coding mRNAs (i.e. 1.94 PAS-clusters per coding mRNA), yielding an average 3′-UTR length of 523 nt (median = 451 nt) (Additional file 10: Table S4), for an example see Figure 7B. Genome-wide we observed that 51.8% genes (n = 1785) contained multiple PAS-clusters compared to 72.1% genes with multiple PASs found in *S. cerevisiae*[35].

Previously, some NATs in *Plasmodium* were shown to be transcribed by RNA polymerase II and it was suggested that NAT stability may be regulated at the level of polyadenylation [36] but no polyadenylation of NATs has been described for *P. falciparum*. Thus, we decided to search for PASs of NATs, i.e. antisense PAS-clusters. Antisense PAS-clusters were defined as those that 1) are located on the antisense strand within the coding region of an annotated gene, 2) contain at least two PASs within the cluster, 3) are located at least 2000 nt upstream of an annotated stop codon (to avoid including polyA tails of neighboring coding mRNA). Because most of the antisense transcripts exist in low abundance, the likelihood of identifying their PASs are much lower than for coding mRNAs. Nonetheless, based on the above criteria, we identified 154 antisense PAS-clusters for 135 genes (for an example see Figure 7C). These data indicate that at least some of the observed NATs are polyadenylated.

### Evidence of antisense spliced junctions

Based on the splicing junctions identified from our datasets using HMMSplicer [37], we found evidence for antisense spliced junctions in 123 of the 1247 genes with NATs (Additional file 11: Table S5). Thus, at least a portion of the observed NATs (~10%) is spliced. Furthermore, we found 55 of the 123 antisense junction clusters to overlap with sense junctions on mRNA (Additional file 11: Table S5). These findings are consistent with a previous study, which has identified antisense introns and antisense junctions overlapping with sense junctions, using a similar approach [6]. These observations further validate antisense transcription in *P. falciparum* to be a widespread phenomenon.

## Discussion

To perform a comprehensive strand-specific analysis of the *P. falciparum* transcriptome, we combined several recent improvements in RNA-Seq technology. Specifically, we took advantage of improvements made in the preparation of highly strand-specific sequencing libraries, we used a polymerase that allows amplification of highly AT-rich DNA sequences and increased the number and length of sequence reads obtained from high-throughput sequencers.

We generated a total of 11 strand-specific transcriptome profiles for parasites across the IDC as well as for separately purified nuclear and cytosolic RNA fractions. Nine of the 11 RNA-Seq libraries were prepared from polyA-enriched RNA because we found these libraries yielded a higher coverage and a more even distribution of sequence reads across the CDSs than libraries prepared from Tex-treated RNA. While polyA-enrichment harbors the intrinsic problem of selecting against non-polyadenylated transcripts, for the following reasons we believe that our libraries contain at least some non-polyadenylated transcripts: 1) PolyA-enrichment is generally not 100% efficient and indeed, tRNA and rRNA sequencing reads were present in our libraries. 2) The *Plasmodium* genome is extremely AT-rich and long stretches of As exist even within genes. Thus it is likely that polyA-enrichment can also enrich for non-polyadenylated transcripts as long as they have internal stretches of As. Nevertheless*,* most likely our dataset lacks weakly transcribed RNA without polyA-tails.

Our analysis revealed transcription from almost 80% of the *Plasmodium* genome and exposed antisense transcription for 24% of genes many of which are developmentally regulated. Interestingly, we find that antisense transcripts are not uniformly distributed but strongly enriched at the 3′-ends of CDSs. Such an enrichment of antisense transcripts at the 3′-end of CDSs has been detected in both yeast and humans [24, 38] and it has been suggested that they can arise via pathways that copy mRNA, from run-through transcription, bi-directional transcription initiation or promiscuous transcription initiation from nucleosome depleted regions (NDRs) [24, 25, 38, 39]. However, the biological function of these 3′-antisense transcripts remains unknown. Our analysis indicates that in *Plasmodium,* for the majority of genes, antisense levels do not correlate with sense transcript levels, which would have hinted at an RNA-copying mechanism or a role of NDRs. In contrast to the corresponding sense mRNA levels, for a large number of genes (37.9%) changes in NATs levels correlate with changes in mRNA levels of the gene located downstream. This correlation may be due to an overlap of different UTRs or of UTRs with CDSs. Such an overlap of UTRs raises the intriguing possibility that transcription of one gene may influence transcription of its neighboring gene and that the genomic location of a gene may be important for its correct regulation. For example, a gene knockout followed by expression of the same gene from a different genomic locus may lead to different antisense transcript levels compared to those from the endogenous locus. In addition, replacement of an endogenous gene with a resistance marker will not only lead to a loss of the mRNA of the endogenous gene but also to a loss of the corresponding antisense RNA, possibly leading to confusing consequences and complicating the interpretation of the results. While our analysis revealed a positive correlation between antisense and sense transcript levels of neighboring genes, such a correlation could not be identified for all genes. Thus, for a significant number of genes (41.6%) our data point to the existence of cryptic promoters at the 3′-end of genes that initiate transcription of NATs independent of the promoter that controls transcription of sense RNA.

Reports on the role of antisense RNA transcripts in *P. falciparum* have been conflicting, some studies found increased antisense levels to repress gene expression [40–44], while others saw no effect [11, 45]. Correlating our RNA-Seq data with previously published proteomics data [32] we find a general correlation between mRNA levels and peptide levels, however, we observed no consistent effect of antisense transcripts on protein levels. Thus it remains to be seen if antisense transcripts modulate gene expression in this parasite.

3′-UTRs and polyA tails have important roles in mRNA localization, stability and regulation of translation (reviewed in [46]). Such regulation can be mediated by *cis*-acting sequence elements that interact with RNA-binding proteins. In *Plasmodium* little is known about how transcription initiation is regulated but numerous studies have reported post-transcriptional control, particularly at the level of translational repression (TR) [47]. Specific sequence motifs within the 3′-UTR have been correlated with TR but genome-wide searches for regulatory motifs have not been performed for a lack of information on 3′ UTRs’ length. In this study we present the genome-wide assignment of PASs in *Plasmodium*. We were able to map PASs for 65% of genes (3443 of 5284), which we believe will represent a valuable resource to the community. For example, the newly generated data should allow other researchers to perform systematic genome-wide searches for sequence motifs located in UTRs that may help elucidate some of the secrets of post-transcriptional gene regulation in the blood stages of *P. falciparum*.

Besides the mapping of mRNA PASs we investigated the presence of antisense PAS-clusters. The latter analysis was much more challenging than the mapping of sense PASs but our data indicate that at least some of the observed antisense transcripts are polyadenylated. This observation further strengthens the assumption that NATs are generated by transcription of DNA and not via transcription of mRNA. This finding is not unexpected given previously published findings that antisense transcription in *Plasmodium* can be carried out by RNA polymerase II (RNA pol II) [36] and that termination of RNA pol II transcription and polyadenylation are commonly coupled. Future deep sequencing projects with libraries derived from RNA fragments enriched for the 3′-end of CDSs should help determine if all NATs are polyadenylated or just represent a small subset.

## Conclusions

Intergenic and non-coding regions of the *P. falciparum* genome have been particularly understudied due to high AT-content, typically ranging between 85 and 95%. Using a protocol that permits the generation of highly strand-specific RNA-Seq libraries and KAPA HiFi DNA polymerase, a polymerase that amplifies even extremely AT-rich DNA, we generated a comprehensive transcriptome dataset for *P. falciparum,* containing great detail about the origin of antisense RNA. Given the large number of independently regulated NATs, our work suggests that ncRNA in *P. falciparum* is of biological significance and not merely a consequence of noisy transcriptional regulation. Importantly, our high coverage and long sequence reads allow us to provide for the first time an extensive list of polyadenylation sites for blood stage parasites. This data should enable others to perform comprehensive searches for regulatory motifs in UTRs and help to understand post-transcriptional regulation of gene expression in *P. falciparum.*

## Methods

### RNA isolation and mRNA enrichment

Parasites from highly synchronous cultures were harvested 10 h, 20 h, 30 h or 40 h post-infection, red bloods cells lysed (0.15% Saponin in PBS) and total RNA (including small RNA) isolated using a miRNeasy Mini Kit (Qiagen). Separation of nuclear and cytosolic RNA fractions was performed as published previously [48]. Subsequently, genomic DNA was removed by on-column DNase treatment according to the manufacturer’s instructions (Qiagen) and mRNA was enriched by subjecting the total RNA to one round of polyA-selection using oligo(dT)-coated Dynabeads (Invitrogen) or treatment with 5′-phosphate-dependent exonuclease (Tex). For polyA-selection (libraries 1-9, Figure 2), we followed the instruction provided by Invitrogen. Alternatively (libraries 10 and 11, Figure 2), 25 μg of total RNA was treated with 10U of Tex (Epicentre), 1x Buffer A (Epicentre), 200U RNase Out (Invitrogen) in a final volume of 200 μl for 60 min at 30°C.

### Strand-specific RNA-Seq library construction

For the preparation of each strand-specific RNA library we used between ~50 ng (ring stage parasites) and ~150 ng (trophozoites) mRNA. mRNA was fragmented to approximately 100-200 nt in length. Reproducible fragmentation was obtained by mixing RNA with a RNA fragmentation reagent (Ambion) and heating it to 70°C for exactly 5 min. To remove the 5′-terminal 7-methylguanylate cap, the fragmented RNA was treated with 10U of Tobacco Acid Pyrophosphatase (Epicentre) for 2 hours at 37°C. All subsequent steps were performed according to an Illumina application Note (Note: directional mRNA-Seq sample preparation) with the following exceptions: Custom-made 5′- adapter (5′-GUUCAGAGUUCUACAGUCCGACGAUCNNNN-3′, conc. 20 μM) was used instead of the RNA adapter provided by Illumina, the PCR amplification was performed for 12-16 cycles using the KAPA HiFi DNA polymerase (Kapabiosystems) and KAPA HiFi Fidelity Buffer according the manufacturer’s instructions (Mg^2^ concentration was adjusted to 2.5 mM). The PCR product was purified and concentrated using AMPure XP beads (Beckman Coulter). Quality and concentration of all libraries was determined using a Bioanalyzer 2100 (Agilent) and high throughput sequencing was performed on a HiSeq2000 (Illumina) except for library 11, Figure 2, which was sequenced on a Genome Analyzer (Illumina). For read statics see Figure 2.

### Genomic DNA library construction

For the preparation of gDNA libraries we fragmented 2.5 μg gDNA to a size of 200-300 bp using Bioruptor (Diagenode). Subsequently, the DNA was blunted using the End-It DNA end-repair kit from Epicentre according to the manufacturer’s instruction and a single dA was added to the 3′ end by mixing the DNA with 5 μl of 10x Buffer 2 (100 mM Tris-HCl, pH 7.9, 500 mM NaCl, 100 mM MgCl_2_, 10 mM DTT, (New England Biolabs)), 10 μl of 1 mM dATP, 15U Klenow fragment (3′-5′ exo^-^) in a final volume of 50 μl followed by an incubation for 30 min at 37°C. Next, a ‘forked DNA adapter’ consisting of DNA strands 5′ P-GAT CGG AAG AGC GGT TC AGCAGGAATGCCGAG-3′ and 5′-ACACTCTTTCCCTACACG ACGCTCTTCCGATct-3′ (phosphodiester bond between c and t) was ligated to the DNA by mixing the DNA with 2.5 μl of 40 μM forked adapter, 25 μl of 2× ligation buffer (Enzymatics), 5 μl of highly purified T4 ligase (600U/μl, Enzymatics) in a total volume of 50 μl followed by incubation of 15 min at 25°C. Ligated DNA ranging in size between 300 and 500 bp was size-selected on an agarose gel and PCR amplified for 12 cycles using the KAPA HiFi DNA polymerase (Kapabiosystems) and KAPA HiFi Fidelity Buffer according to the manufacturer’s instructions (Mg^2^ concentration was adjusted to 2.5 mM). The PCR product was purified and concentrated using AMPure XP beads (Beckman Coulter). Quality and concentration was determined using a Bioanalyzer 2100 (Agilent) and high-throughput sequencing was performed on a HiSeq2000 (Illumina). Between enzymatic reaction the DNA was purified using NucleoSpin Extract II columns (Macherey-Nagel).

### Mapping of sequence reads

First, reads longer than 50 nt flagged with Illumina’s low quality flag “B” were removed from all datasets. Then, we removed the 4 custom index nucleotides at the 5′-end and the low quality nucleotides at the 3′-end of the reads. The *P. falciparum* 3D7 genome and its gene model annotations (version 3 on January 2012) were downloaded from Sanger FTP (ftp://ftp.sanger.ac.uk/pub/pathogens/Plasmodium/falciparum/). Unless specified otherwise, the reads in all datasets were mapped onto the reference genome using Bowtie 0.12.8 (parameters “-n 2 -k 1 -m 50 --best”), with maximum 2 mismatches and multiple hits (maximum 50) distributed to the best-aligned location [49]. To map the splicing junctions, the unaligned reads from Bowtie were mapped using HMMSplicer (parameters “-w 4 -j 10 -k 3000 -ja 10 -e 2 -m 500 -n 700 -d True”). It should be noted that HMMSplicer was specifically developed to work with *P. falciparum* RNA-Seq data [37]. Read pileups were generated for each library and all read pileups (n = 11) were pooled using custom scripts to generate a coverage map for defining the genomic distribution of NATs. Strand-specific fastq data of López-Barragán et al. [7] were downloaded from Short Read Archive of NCBI (http://www.ncbi.nlm.nih.gov/sra/) under the accession of SRR364836, SRR364841, SRR364842 and SRR364846. Second reads of the pairs in these datasets were reverse complemented and all reads were processed in the same way as our datasets (mentioned above).

### Transcript expression levels and their fold changes across IDC

The expression level of a transcript was expressed as number of reads per kilobase per million (RPKM) [50]. Briefly, we counted the number of reads mapped to all annotated transcriptomic features (e.g. mRNA) on the same strand (i.e. sense) and opposite strand (i.e. antisense). Both the sense and antisense read numbers were normalized by the length of the feature (in kilobase) and the total number of reads (in millions) mapped to non-structural RNAs in the corresponding library (i.e. number of mappable reads excluding rRNA and tRNA reads). To visualize the changes of sense and antisense expression levels across the 4 time points across the IDC, we generated expression heat maps based on both sense and antisense RKPM using Genesis (http://genome.tugraz.at/). Briefly, the order of genes was sorted based on the changes of sense RKPM (in log2 scale) across the 4 time points, and a heat map was generated using both sense and antisense RKPM while keeping the same gene order. The process was then repeated using antisense RKPM for gene sorting. The patterns of these heat maps were then visually inspected as described in the main text. To identify transcripts that are developmentally regulated, we used EdgeR [51] to screen for genes that are differentially expressed across the IDC. Briefly, each gene was assigned to have a sense and antisense transcript of the same length (i.e. coding region), which were then treated as independent transcripts in the EdgeR analyses. The read counts on these transcripts among the 4 time points were paired into 6 non-redundant pairs and compared using EdgeR with the biological coefficient of variation set to 0.6. We then used the exact test for determining differential expression and transcripts at false discovery rate (FDR) < 0.05 were considered to be differentially expressed. A transcript is considered developmentally regulated if it is differentially expressed in at least 2 of the 6 non-redundant time point pair comparisons.

### Prevalence and coverage pattern of natural antisense transcripts

To determine the prevalence of natural antisense transcripts, we pooled the reads from all 4 time-points (i.e. library 1 to 4) and generated a strand-specific read coverage map. Based on this map, we scanned for transcribed fragments (transfrags) longer than 150 nt covered by at least 2 fold-coverage per nt with a maximum 10nt of coverage gap. A transfrag will be split into shorter transfrags if a dramatic difference in coverage is detected within a sliding window (≥100 fold difference between two halves of a 20 nt window). An antisense transfrag is then defined as a transfrag that is overlapping with the CDS of a gene in the opposite strand. A significant antisense transfrag is defined based on the following 4 criteria 1) percentage of ORF covered by the transfrag: ≥10% (stringent criteria) or ≥5% (relaxed criteria), 2) average coverage depth of the transfrag: ≥10 (stringent) or ≥3 (relaxed), 3) antisense read count: ≥50 (stringent) or ≥15 (relaxed), and 4) sense to antisense read ratio: <200 (stringent) or <2000 (relaxed). A significant sense transfrag was defined in a similar way except for criteria 4). To investigate the overall coverage pattern of antisense (or sense) transfrags (at stringent cutoffs), we divided the CDSs into 5 equal bin regions and recorded the overlap of antisense (or sense) transfrags within these bin regions. The percentage of genes being covered by antisense (or sense) transfrags in these bin regions was plotted (as shown in Figure 4A).

### Detection of potential run-through transcription and potential bi-directional promoters

We reasoned if an antisense transcript observed in a gene was the consequence of the transcription activities from its downstream gene through a bi-directional promoter (CDSs are in ‘Tail-to-Head’ orientation) or run-through transcription (CDSs are in ‘Tail-to-Tail’ orientation), changes in expression level of the upstream antisense transcript across IDC should be positively correlated to that of the downstream mRNA. We therefore investigated the correlation between the changes in antisense transcript level of the upstream gene (upStrmA RPKM) and the changes in sense transcript (i.e. mRNA) level of the downstream gene (dnStrmS RPKM) across the 4 IDC time-points. Briefly, we calculated the fold changes of both sense and antisense RPKM for each gene among the 4 time points (i.e. 6 non-redundant pairs of time points). A valid comparison requires either 1) both RPKM values are ≥0.2 in both time points (i.e. quantifiable), or 2) one RPKM value is ≥2 if the other RPKM value is <0.2 (i.e. unquantifiably large). In a comparison, if both upStrmA RPKM and dnStrmS RPKM were changed in the same direction at ≥1.5 fold, this comparison is scored as “positive”, and alternatively, as “negative” if the change was in opposite direction, or otherwise, as “not correlated”. A comparison could also be scored as “no data” if the RPKM values were lower than the mentioned cutoff. Based on the scores of these 6 comparisons, the correlation of upStrmA RPKM and dnStrmS RPKM in a gene pair is said to be “positive (strong)”, “positive (medium)” or “positive (weak)” if “positive” is scored in ≥3, ≥2 and ≥1 of the 6 comparisons and no “negative” was scored. Negative correlations were defined in the same manner but in the opposite direction. Correlations of a gene pair were defined as “not correlated” in other scenarios, e.g. both “positive” and “negative” were scored, or as “no data” if all 6 scores were “no data”. Detailed results can be found in Additional file 6: Table S3. Finally, strength of evidence supporting a bi-directional promoter affecting its upstream antisense transcript was defined as strong, medium and weak when the correlation of a Tail-to-Head gene pair is “positive (strong)”, “positive (medium)” and “positive (weak)”, respectively. Strength of evidence supporting run-through transcription affecting the upstream antisense transcript is defined in the same manner except that Tail-to-Tail gene pairs were considered (Additional file 6: Table S3). It should be noted that 181 coding genes were not analyzed because they do not have an annotated coding gene at their 3′-end, e.g. located at chromosome end, or next to pseudogene, tRNA or rRNA, etc. Totally, 2774 Tail-to-Tail and 2329 Tail-to-Head pairs were analyzed.

### Assessment of repetitiveness using simulated genomic DNA reads

Interpretation of differences in alignment patterns observed for uniquely and non-uniquely mapped reads to repetitive regions of the genome is difficult, if the repetitiveness of these regions was unknown. We therefore assessed the repetitiveness of various genomic regions by simulation. Briefly, we randomly extracted 10 million ‘fragments’ from both strands of the whole genome (mean 200 nt with standard deviation 50 nt). Then, we extracted the first 60 nt from these simulated ‘fragments’ as simulated ‘reads’. These simulated reads were mapped to the genome using Bowtie with zero mismatch allowed and maximum alignment hit of 100. Then, we defined the repetitiveness of a position in the genome as the averaged number of alignment hits (i.e. NH attribute of SAM file specification) of all the reads mapped to this position. By definition, repetitiveness of 1 means all reads mapped to this position are uniquely mapped reads. This ‘repetitiveness’ value was used in Figure 3.

### Identifying the polyadenylation sites from reads

The criteria used here are primarily based on Lee et al. [33]. Briefly, reads containing 15 or more consecutive “A” at their 3′-end were selected from all datasets and redundant reads within the same library were discarded. These non-redundant reads were pooled. These reads potentially contain the sequence of polyA tails. The A stretches at the end were trimmed and the reads with minimal 18 nt after trimming were mapped to the reference genome using Bowtie with parameters “-n 2 -k 1 -m 50 -1 30”. To distinguish polyA tracks of true polyadenylation from polyA tracks of internal polyA stretches on the mRNAs themselves (i.e. false positives), we analyzed the base compositions surrounding the end of the mapped reads and discarded those that might not represent true polyadenylation. Reads with the following properties were regarded as false positives and removed. 1) Reads with ≥ 5 nt immediate downstream of the end site are As; 2) Depending on the actual length of the polyA stretch of the read (e.g. N nt), reads with 70% of N nt downstream of the end site are As; 3) Reads with ≥ 6nt within 10 nt immediate upstream of the end site are As. The polyadenylation sites were then defined as the immediate downstream base of the reads. To ensure the identified polyadenylation site are not false positives derived from low quality base calls, reads with quality scores in any of the upstream and downstream 5 nt flanking the polyadenylation site less than 20 were further removed. These procedures should be able to remove false positives derived from internal polyA stretches and low quality base calls.

### Assigning the polyA site clusters to gene models

As most of the observed polyadenylation sites appear as clusters [34], we grouped the polyA sites into clusters by allowing an optimal maximum intra-cluster distance (at 20 nt) between sites. A polyA cluster was then represented by the polyA site with the highest number of supporting reads (i.e. peak), and these peak positions were used in all downstream analyses. A polyA cluster is defined as valid when the number of reads at the peak position is ≥ 2. To assign polyA tails to mRNAs, we searched for polyA clusters within 2000 nt downstream of their stop codons on the same strand and recorded the size of the coverage gap between the polyA clusters and the stop codon. A polyA tail is defined as valid when the coverage gap is ≤ 30 nt.

### Analysis of antisense splicing junctions

All splicing junctions identified by HMMSplicer [37] were clustered as mentioned in a previous study [52]. A junction cluster is considered to be ‘antisense’ when its representative junction is located within the coding region of a gene on the opposite strand.

### Availability of supporting data

All sequence reads from this study have been submitted to the EMBL Nucleotide Sequence Database (EMBL-Bank) (http://www.ebi.ac.uk/embl/) under accession no. ERP001849.

## Electronic supplementary material

Additional file 1: Figure S1: Percentage versus coverage of the transcribed *P. falciparum* genome. Levels of transcription are based on the combined data from 11 libraries. (PDF 95 KB)

Additional file 2: Figure S2: Comparison of pooled datasets from López-Barrágan et al. (4 libraries) and this study (11 libraries). **A)** Coverage of dataset. Venn diagram showing the percentage of the genomic nucleotides being covered in the two datasets as indicated (≥ 5-fold coverage in either strand). **B)** Correlation of sense and antisense reads mapped to coding genes. Each dot represents a gene. X-axis and y-axis refer to number of reads mapped to the corresponding genes in sense and antisense strand, respectively. Left and right panel: pooled dataset of this study and López-Barragán et al. respectively. Pearson’s correlations of the plots are indicated. A globally positive correlation has been used as an indicator for potential artifactual antisense transcription. (PDF 931 KB)

Additional file 3: Table S1: Table listing sense and antisense transcript level for all genes across the IDC. (XLSX 889 KB)

Additional file 4: Table S2: Table listing data from the EdgeR-based analysis of developmental regulation of sense and antisense transcripts. (XLSX 633 KB)

Additional file 5: Figure S3: Changes in sense and antisense transcript levels are not correlated. Scatter plot of fold-changes in antisense RPKM versus fold-changes in sense RPKM between 4 pairs of time points (10-20 h, 20-30 h, 30-40 h and 40-10 h, n = 7600). Data is plotted only for genes with both sense and antisense RPKMs values ≥0.5. (PDF 138 KB)

Additional file 6: Table S3: Correlation of antisense RNA levels with mRNA levels of neighboring gene. (XLSX 633 KB)

Additional file 7: Figure S4: Antisense transcripts are not globally accumulated in the nucleus. Relative abundance, which is calculated as reads per kilobase per million (RPKM) (see Materials and methods), reflects the proportion of a transcript in libraries from different RNA fractions. As mRNA consists >90% of all non-ribosomal RNA reads in all libraries and assuming the overall proportion of mRNA does not vary significantly between libraries, relative abundance can thus be used as an indicator to measure whether the overall proportion of the less abundant transcripts, e.g. subtelomeric transcripts and antisense transcripts, varies significantly between libraries. **A)** Relative abundance of subtelomeric transcripts (n = 10) is significantly higher (P < 0.001 in Student’s t-test) in the nuclear library than in both total and cytosol libraries. **B)** Relative abundance of antisense transcripts (n = 198) is not significantly different between nuclear, cytosol and total libraries. Genes used for these comparisons have significant levels of antisense transcription (see text), are not likely to be affected by run-through transcription from neighboring genes (see Additional file 3: Table S1) and have non-zero antisense RPKM values in all three libraries. All data was generated based on libraries of 20 h p.i. Asterisks, P < 0.001 in Student’s t-test. The “boxes and whiskers” represent the 5th, 25th, 50th, 75th and 95th percentiles. (PDF 127 KB)

Additional file 8: Figure S5: Correlation of transcript levels with peptide levels. **A)** Scatter plot showing sense sequence reads per Kb versus peptide counts per Kb for genes with at least one sequence read and one peptide count. **B)** Scatter plot showing antisense sequence reads per Kb versus peptide counts per Kb for genes with at least one sequence read and one peptide count. Peptide count data was taken from Le Roch et al. [35]. (PDF 791 KB)

Additional file 9: Figure S6: Evaluation of AT-bias in library preparation. (PDF 127 KB)

Additional file 10: Table S4: Table listing predicted polyadenylation sites. (XLSX 423 KB)

Additional file 11: Table S5: Table listing NAT splice junctions. (XLSX 278 KB)
