# Supplementary figures and images for: Strand-specific RNA-Seq reveals widespread and developmentally regulated transcription of natural antisense transcripts in Plasmodium falciparum
Source: BMC Genomics. 2014 Feb 22;15(1):150. doi: 10.1186/1471-2164-15-150 (PMC4007998; doi:10.1186/1471-2164-15-150)

Supplementary Figure S1

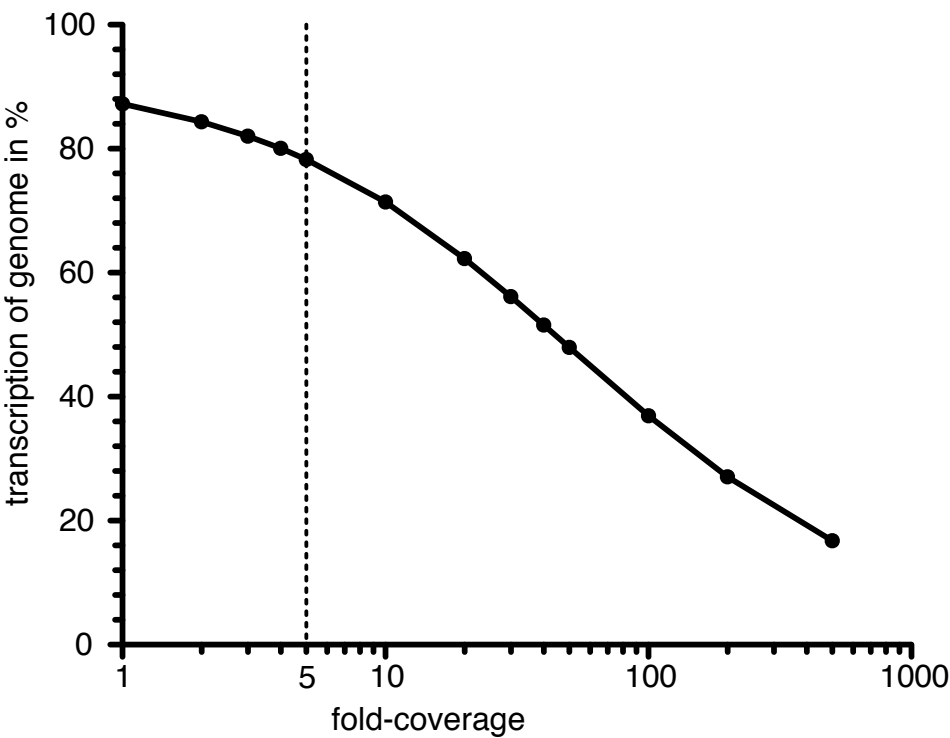

Supplement: Supplementary file 1 — Additional file 1: Figure S1: Percentage versus coverage of the transcribed P. falciparum genome. Levels of transcription are based on the combined data from 11 libraries. (PDF 95 KB) [file 12864_2013_7013_MOESM1_ESM.pdf]

# Supplementary Figure S2

(a) Percentage of genome being transcribed

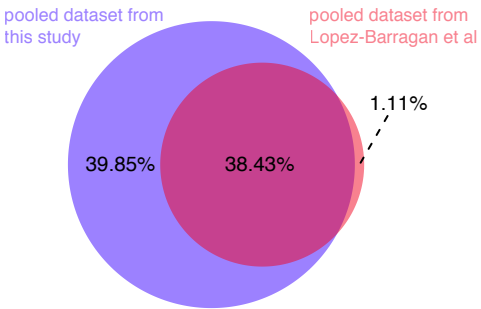

(b)

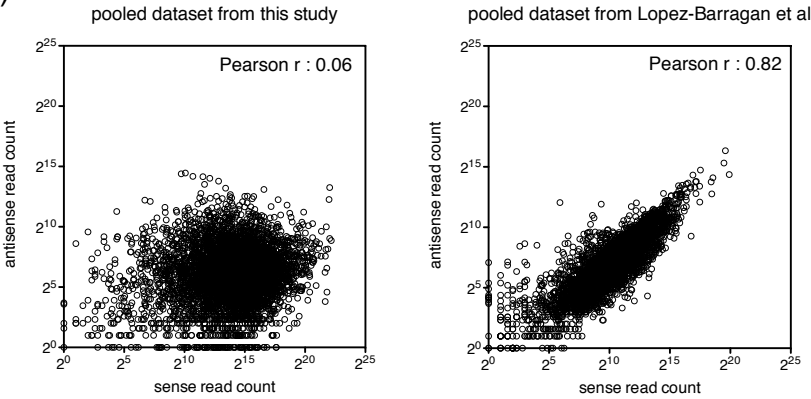

Supplement: Supplementary file 2 — Additional file 2: Figure S2: Comparison of pooled datasets from López-Barrágan et al. (4 libraries) and this study (11 libraries). A) Coverage of dataset. Venn diagram showing the percentage of the genomic nucleotides being covered in the two datasets as indicated (≥ 5-fold coverage in either strand). B) Correlation of sense and antisense reads mapped to coding genes. Each dot represents a gene. X-axis and y-axis refer to number of reads mapped to the corresponding genes in sense and antisense strand, respectively. Left and right panel: pooled dataset of this study and López-Barragán et al. respectively. Pearson’s correlations of the plots are indicated. A globally positive correlation has been used as an indicator for potential artifactual antisense transcription. (PDF 931 KB) [file 12864_2013_7013_MOESM2_ESM.pdf]

Supplemental Figure S4

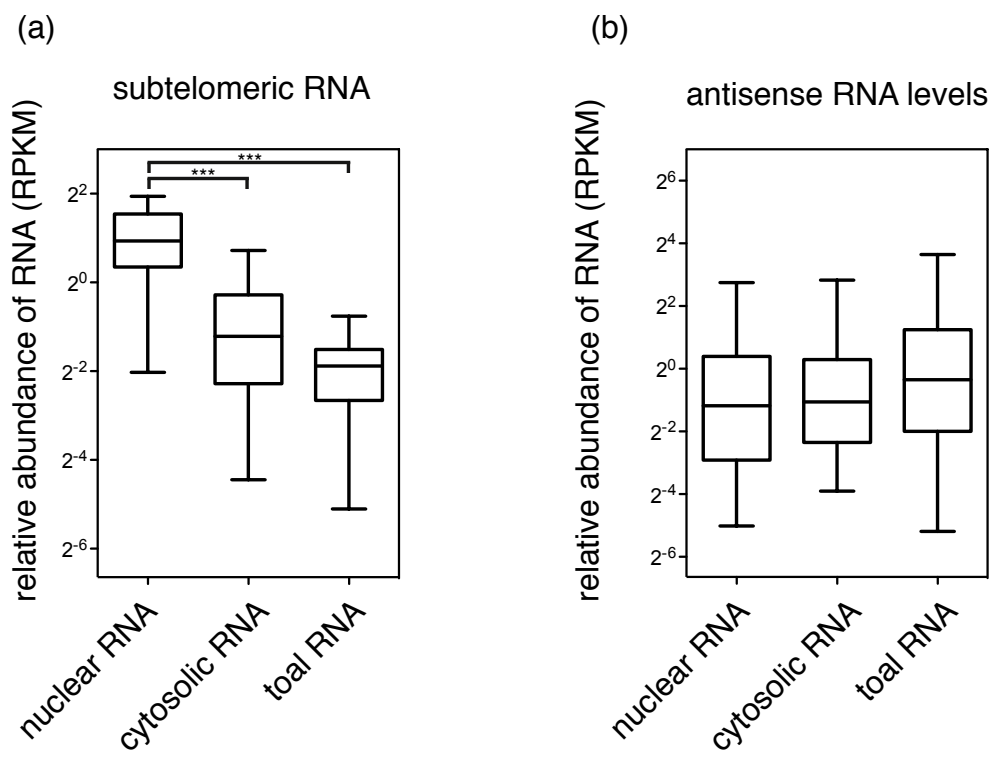

Supplement: Supplementary file 5 — Additional file 5: Figure S3: Changes in sense and antisense transcript levels are not correlated. Scatter plot of fold-changes in antisense RPKM versus fold-changes in sense RPKM between 4 pairs of time points (10-20 h, 20-30 h, 30-40 h and 40-10 h, n = 7600). Data is plotted only for genes with both sense and antisense RPKMs values ≥0.5. (PDF 138 KB) [file 12864_2013_7013_MOESM5_ESM.pdf]

Supplementary Figure S6

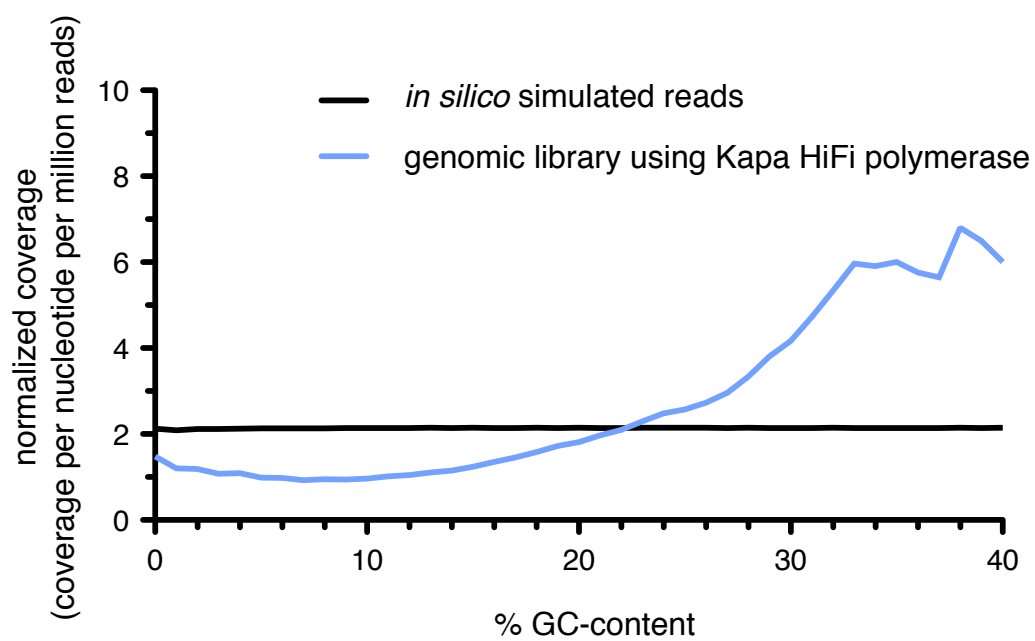

Supplement: Supplementary file 7 — Additional file 7: Figure S4: Antisense transcripts are not globally accumulated in the nucleus. Relative abundance, which is calculated as reads per kilobase per million (RPKM) (see Materials and methods), reflects the proportion of a transcript in libraries from different RNA fractions. As mRNA consists >90% of all non-ribosomal RNA reads in all libraries and assuming the overall proportion of mRNA does not vary significantly between libraries, relative abundance can thus be used as an indicator to measure whether the overall proportion of the less abundant transcripts, e.g. subtelomeric transcripts and antisense transcripts, varies significantly between libraries. A) Relative abundance of subtelomeric transcripts (n = 10) is significantly higher (P < 0.001 in Student’s t-test) in the nuclear library than in both total and cytosol libraries. B) Relative abundance of antisense transcripts (n = 198) is not significantly different between nuclear, cytosol and total libraries. Genes used for these comparisons have significant levels of antisense transcription (see text), are not likely to be affected by run-through transcription from neighboring genes (see Additional file 3: Table S1) and have non-zero antisense RPKM values in all three libraries. All data was generated based on libraries of 20 h p.i. Asterisks, P < 0.001 in Student’s t-test. The “boxes and whiskers” represent the 5th, 25th, 50th, 75th and 95th percentiles. (PDF 127 KB) [file 12864_2013_7013_MOESM7_ESM.pdf]

# Supplementary Figure S5

(a)

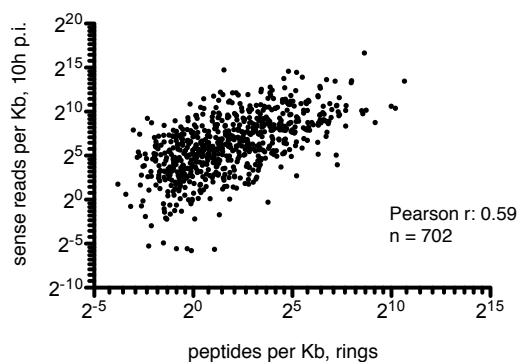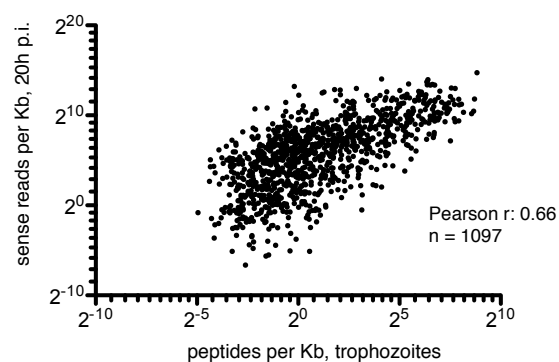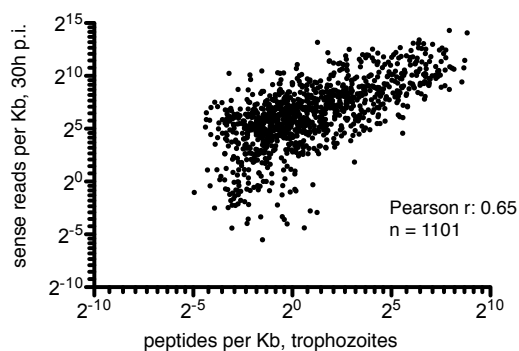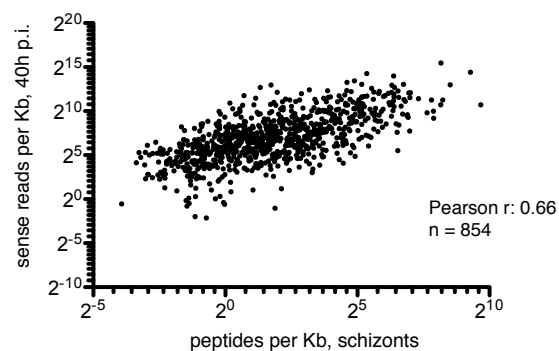

(b)

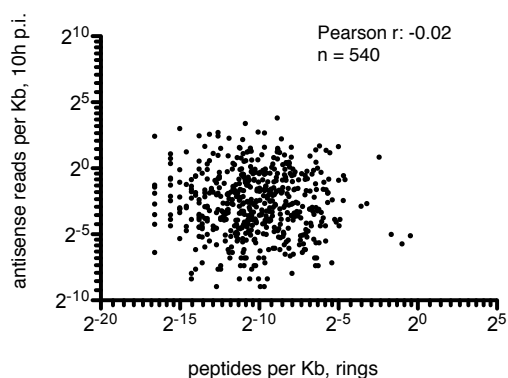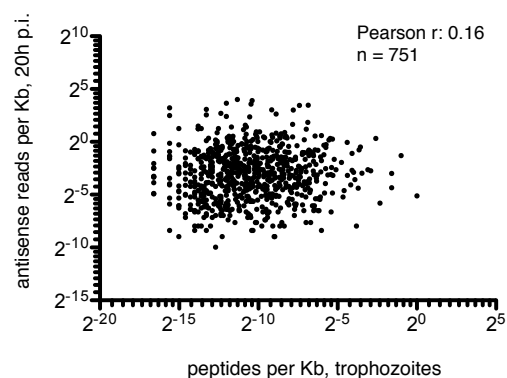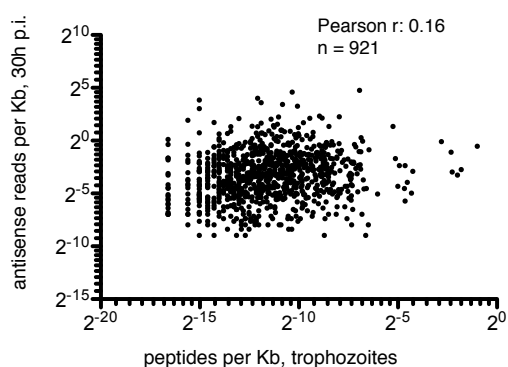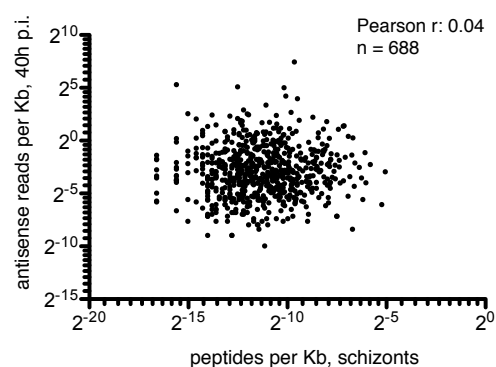

Supplement: Supplementary file 8 — Additional file 8: Figure S5: Correlation of transcript levels with peptide levels. A) Scatter plot showing sense sequence reads per Kb versus peptide counts per Kb for genes with at least one sequence read and one peptide count. B) Scatter plot showing antisense sequence reads per Kb versus peptide counts per Kb for genes with at least one sequence read and one peptide count. Peptide count data was taken from Le Roch et al. [35]. (PDF 791 KB) [file 12864_2013_7013_MOESM8_ESM.pdf]
